# The relationship between publication of high-quality evidence and changes in the volume and trend of subacromial decompression surgery for patients with subacromial pain syndrome in hospitals across Australia, Europe and the United States: a controlled interrupted time series analysis

**DOI:** 10.1186/s12891-023-06577-6

**Published:** 2023-06-03

**Authors:** Timon H. Geurkink, Leti van Bodegom-Vos, Jochem Nagels, Susan Liew, Pieter Stijnen, Rob G.H.H. Nelissen, Perla J. Marang-van de Mheen

**Affiliations:** 1 Department of Orthopaedics, Leiden University Medical Centre, Postbus, Leiden, 9600, 2300 RC the Netherlands; 2 Department of Biomedical Data Sciences, Medical Decision Making, Leiden University Medical Centre, Leiden, the Netherlands; 3 Department of Orthopaedic Surgery, Alfred Hospital, Melbourne, Australia; 4 Department of Management Information and Reporting, University Hospital Leuven, Leuven, Belgium

**Keywords:** Subacromial pain syndrome, Subacromial decompression, Interrupted time series, Segmented regression, Low-value care

## Abstract

**Aims:**

To evaluate the extent to which publication of high-quality randomised controlled trials(RCTs) in 2018 was associated with a change in volume or trend of subacromial decompression(SAD) surgery in patients with subacromial pain syndrome(SAPS) treated in hospitals across various countries.

**Methods:**

Routinely collected administrative data of the Global Health Data@work collaborative were used to identify SAPS patients who underwent SAD surgery in six hospitals from five countries (Australia, Belgium, Netherlands, United Kingdom, United States) between 01/2016 and 02/2020. Following a controlled interrupted time series design, segmented Poisson regression was used to compare trends in monthly SAD surgeries before(01/2016-01/2018) and after(02/2018-02/2020) publication of the RCTs. The control group consisted of musculoskeletal patients undergoing other procedures.

**Results:**

A total of 3.046 SAD surgeries were performed among SAPS patients treated in five hospitals; one hospital did not perform any SAD surgeries. Overall, publication of trial results was associated with a significant reduction in the trend to use SAD surgery of 2% per month (Incidence rate ratio (IRR) 0.984[0.971–0.998]; *P* = 0.021), but with large variation between hospitals. No changes in the control group were observed. However, publication of trial results was also associated with a 2% monthly increased trend (IRR 1.019[1.004–1.034]; *P* = 0.014) towards other procedures performed in SAPS patients.

**Conclusion:**

Publication of RCT results was associated with a significantly decreased trend in SAD surgery for SAPS patients, although large variation between participating hospitals existed and a possible shift in coding practices cannot be ruled out. This highlights the complexities of implementing recommendations to change routine clinical practice even if based on high-quality evidence.

**Supplementary Information:**

The online version contains supplementary material available at 10.1186/s12891-023-06577-6.

## Introduction

The subacromial pain syndrome(SAPS) is an umbrella diagnosis that accounts for up to 70% of cases with shoulder pain [1]. Although most SAPS patients are treated non-operatively, a substantial part undergoes subacromial decompression(SAD) surgery [2]. High-quality randomised controlled trials(RCTs), however, found no significant improvement in pain or function after SAD surgery in SAPS patients compared with nonoperative management and placebo surgery [3–12]. Moreover, SAD surgery carries a risk of harm for patients and contributes to increased resources [11, 13]. Therefore, SAD surgery for SAPS is considered low-value care, a term used to refer to treatment or tests where there is little or no benefit for patients or more potential harm than benefit, and a strong international recommendation has been formulated against its use [14]. Multiple studies previously investigated trends in worldwide use of SAD surgery for SAPS [13, 15–21]. Decreasing trends have been reported in various countries, such as the Netherlands, Finland, Scotland and the United States(US), but increasing trends were observed in Australia, the United Kingdom(UK) and the US [13, 15–22]. No studies have examined trends in SAD surgery beyond 2017, whereas two high-quality RCTs were published in 2018 that may have impacted routine clinical practice [4, 6].

Exploring how publication of high-quality evidence may influence clinical decision-making in routine clinical practice has received limited attention in orthopaedic literature [23]. Timely implementation of evidence is of vital importance for both healthcare providers and patients, as performing low-value procedures does not provide the patient with the best treatment and contributes to rising healthcare costs [24]. The studies by Beard- and Paavola et al. were the first two placebo-controlled trials and formed the foundation for the strong international recommendation against SAD surgery by a panel assembled by the British Medical Journal [14]. It is, however, unknown to what extent publication of these RCTs has changed previous trends in SAD surgery in daily practice. Therefore, the aim of this study was to evaluate the extent to which publication of these high-impact RCTs in 2018 was associated with changes in the absolute volume or trend in monthly SAD surgeries in hospitals from different countries.

## Methods

### Study design

A controlled interrupted time series(ITS) design was used, which is a powerful quasi-experimental approach to evaluate effects of an intervention implemented at a clearly defined time point [25–29] and previously shown to give concordant results as those from a cluster RCT [30]. By comparing the trend before and after intervention, the intervention effect can be estimated by a change in absolute level and/or change in trend [26]. A change in trend represents a gradual change in daily practice following an intervention, whereas a change in level constitutes a more abrupt effect [31]. Given the importance of the two trials published in 2018 for subsequent recommendations, we used the publication month of the first published RCT(01/2018) as the intervention time. We compared the volume of monthly SAD surgeries before(01/2016–01/2018), with that after the intervention(02/2018–02/2020).

Pseudonymised patient data from the Global Heath Data@Work (GHD@Work) collaborative were used, in which hospitals from various countries share their experiences and compare their outcomes using routinely collected administrative admission data. Data on clinical admissions and day case surgeries) were used for patients from six hospitals in five countries (Australia, Belgium, Netherlands, UK, US). Participating hospitals (appendix A) are large academic medical centres, that are likely comparable with regard to their (complex) patient population. Within the collaborative, diagnoses and procedures were combined into groups and comorbidities in the Elixhauser comorbidity index [32], which were matched across countries to reconcile the different coding systems being used, as done in previous studies [33].

### Patients and definitions

The study population included all patients aged 18 + years with a primary or secondary diagnosis potentially indicating SAPS, who underwent surgery in participating hospitals between 01/2016 and 02/2020. We excluded data from 03/2020 onwards as the number of surgeries was likely affected by the COVID-19 pandemic which would violate one of the key assumptions for the ITS (i.e. the intervention occurred independently of other changes over time) [26]. SAD procedures were identified using a combination of diagnosis and procedure codes. First, all clinical patient admissions and day case surgeries with a possible SAPS diagnosis were selected based on their primary or secondary diagnosis, using the following ICD-10 codes: M75.1-Rotator Cuff Syndrome, M75.2-Bicipital Tendinitis, M75.3-Calcific tendinitis of shoulder, M75.4-Impingement syndrome of shoulder, M75.5-Bursitis of shoulder. Within this patient selection, we selected those with SAD procedure codes. As hospitals from different countries used different coding systems for procedures, these were harmonized across countries to reconcile the differences between coding systems used. To ensure that we would capture local coding practices, we asked experts from participating hospitals to verify the diagnostic and procedure codes that were used to identify this patient group before seeing the results, or that some codes were not used, incorrect or missing (Appendix B).

As control group, we included all other patients likely to be treated by orthopaedic surgeons for musculoskeletal problems to control for potential confounding effects (e.g. other interventions/events occurring during the study period affecting surgery volumes such as a new hospital policy) [34]. The control group was represented by all patients who underwent a procedure within the ICD-10 clusters ‘Diseases of the musculoskeletal system and connective tissue disease’ or ‘Injury and poisoning’ (MSK clusters; Appendix B), excluding SAPS patients, as these clusters will capture most musculoskeletal patients.

It is possible that a change in performed SAD surgeries is accompanied by a shift towards other procedures, either a true change or merely in coding practice among clinicians, for instance if they have strong beliefs that SAD surgery may benefit their patients. Therefore, a sensitivity analysis was carried out to examine changes in performed procedures within the following groups: (1) Any other performed orthopaedic procedure in SAPS patients (SAPS–Other procedures) reflecting a possible shift in procedure coding. Since patients with SAPS as a secondary diagnosis could undergo procedures to treat e.g. cardiac comorbidity, we only included patients within the beforementioned MSK clusters. (2) SAD surgeries in patients with any other diagnosis code than SAPS (NonSAPS–SAD) reflecting a possible shift in diagnosis coding.

### Statistical analysis

First, monthly volumes of admissions and procedures were examined for every hospital to gauge the size of the hospital and the musculoskeletal department for (1) all patients, (2) patients within the MSK clusters, and (3) volume of procedures. Parametric continuous data were described using means, standard deviation (SD) and 95% confidence intervals (CI) and nonparametric data were expressed in medians and interquartile ranges. Categorical data were presented by numbers and percentages.

A segmented Poisson regression model with random intercept for hospital was used to assess changes in level and/or trend of monthly volume of SAD surgeries before (25 Data points) and after (25 Data points) publication of the first RCT [31]. A separated controlled design was used to compare the intervention group with the control group [34]. The same analysis was done for each individual hospital and for the sensitivity analyses. The Kolmogorov-Smirnov test was used to ensure our data followed a Poisson distribution, and robust standard errors to safeguard against any mild violations of regression assumptions [35]. Negative binomial regression was used for over-dispersed count data.

The following equation was used to estimate the changes in level and/or trend associated with publication of the high-quality RCTs (the intervention): Y_t_ = β_0_ + β_1_*Time(months) + β_2_*Intervention + β_3_*Time after intervention + e_t_. Here, Y_t_ is the number of procedures, β1 estimates the pre-intervention trend, while β2 estimates the change in level directly following the intervention and β3 indicates the change in trend following the intervention. A random intercept was included to take into account between-hospital differences in the volume of surgeries, reflecting e.g. different hospital size.

We evaluated stationarity using the augmented Dicky-Fuller and KPSS tests, tested for first order autocorrelation using the Durbin-Watson test and higher order autocorrelations and/or seasonality using (partial) autocorrelation function plots. In case of non-stationarity, data were differenced. No autocorrelation or seasonality was found in the time series. Stata Version 17.1 (StataCorp LLC, USA) was used for analysis. Significance was established at *P* < 0.05.

## Results

Hospital monthly volumes in patients and procedures are presented in Table 1. A total of 3.046 patients undergoing SAD procedures in six hospitals across five countries were included, with 1.601 performed before and 1.445 after publication of the RCTs. One hospital did not perform any SAD surgeries during this period and thereby did not contribute to further analysis. Characteristics of patients undergoing SAD surgery are shown in Table 2, showing considerable variation across hospitals. For instance, patients were older in one US hospital, whereas patients less often had comorbidities and were less often treated in day case surgery in the Australian hospital. The readmission rate varied between 0.1% and 4.4%.

**Table 1:** Hospital monthly volumes of patients* and procedures between 01/2016–02/2020

	*Australia*	*Belgium*	*The Netherlands*	*United Kingdom*	*United States (1)*	*United States (2)*
Median monthly volume of patients (IQR)	9.448 (9.072–9.779)	10.251 (10.046–10.634)	3.641 (3.532–3.797)	12.398 (11.968–12.880)	15.835 (14.618–16.370)	5.646 (5.272–5.967)
Median monthly volume of patients within MSK clusters** (IQR)	1.842 (1.773–1.907)	1.160 (1.110–1.235)	345 (326–364)	1.703 (1.644–1.780)	2.530 (2.383–2.616)	684 (634–750)
Median monthly volume of procedures (IQR)	7.882 (7.628–8.133)	6.705 (6.492–7.082)	948 (903–999)	8.963 (8.559–9.344)	6.788 (6.453–7.064)	2.399 (2.302–2.480)
Median monthly volume of procedures within MSK clusters** (IQR)	1.464 (1.419–1.538)	1.039 (991–1104)	145 (135–155)	1.348 (1.305–1.417)	1.221 (1.145–1.291)	591 (550–616)
Median monthly volume of subacromial decompressions (IQR)	3 (1–4)	17 (12–24)	0	22 (18–27)	1 (0–1)	17 (14–21)

*Includes clinical admissions and day case surgeries. **Monthly volume of patients within the ICD-10 clusters: ‘Diseases of the musculoskeletal system and connective tissue disease’ and ‘Injury and poisoning’, capturing most musculoskeletal (MSK) patients. Abbreviations: IQR = interquartile range.

**Table 2:** Characteristics of patients undergoing subacromial decompression*

	*Australia* (n = 145)	*Belgium* (n = 907)	*United Kingdom* (n = 1102)	*United States (1)* (n = 45)	*United States (2)* (n = 847)
Mean age (SD)	58,8 (11,9)	55,9 (10,6)	57,2 (10,5)	62,3 (12,2)	55,8 (12,8)
% Female	48,3%	56,3%	48,9%	57,8%	40,4%
*Comorbidities* **					
_% ≥1 Comorbidities	23,4%	56,9%	65,7%	57,8%	53,0%
_% Diabetes Mellitus	9,0%	6,2%	13,4%	17,8%	10,7%
_% Hypertension	2,8%	19,6%	27,9%	44,4%	32,1%
_% Obesity	0%	37,2%	35,6%	8,9%	5,8%
_% Pulmonary	0,7%	5,1%	15,3%	13,3%	10,3%
Median number of comorbidities (IQR)	0 (0–0)	0 (0–1)	0 (0–1)	1 (0–2)	0 (0–1)
Median LOS (IQR)	1 (1–1)	1 (0–1)	0 (0–0)	2 (0–7)	0 (0–0)
% Day case surgeries	1,4%	50,6%	85,7%	46,7%	100%
Readmission rate***	2,8%	3,1%	2,5%	4,4%	0,1%

*No subacromial decompressions were performed in the Dutch hospital. ** According to the Elixhauser Comorbidity index, only the most prevalent comorbidities are shown. ***Readmission rate within 30 days after discharge. Abbreviations: SD = Standard Deviation, LOS = Length of Stay, IQR = Interquartile Range.

**Fig. 1:**
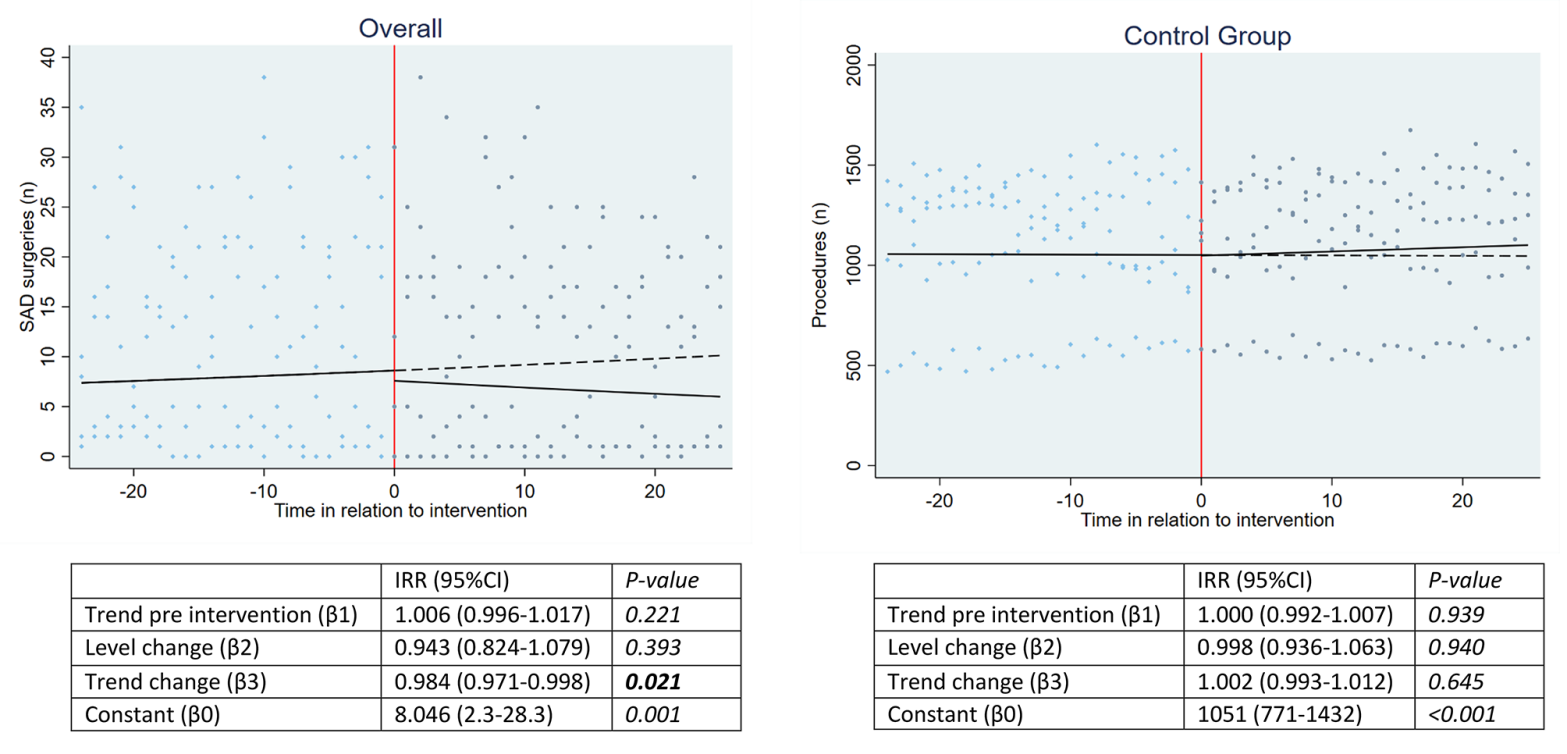
Outcomes overall SAPS group and control group. Shows the fitted trend lines and regression coefficients, adjusted for clustering of patients within hospitals, of the number of monthly SAD surgeries (left; SAPS group) and other orthopaedic procedures (right; control group) before and after publication of the RCTs (time in relation to intervention represented in *months*). The dashed lines represent the fitted trend lines post-intervention as if the RCTs had not been published. Significant values are presented in bold. Abbreviations: SAD = subacromial decompression, IRR = incidence rate ratio, 95%CI = 95% Confidence Interval

Figure 1 shows wide variation in volume of SAD surgeries (indicated by the data points), reflecting the different size of hospitals and/or musculoskeletal departments. Adjusting for clustering of patients within hospitals, there was no significant trend in volume of SAD surgeries before publication of the RCTs (Incidence rate ratio (IRR):1.006[0.996–1.017]; *P =* 0.221). Publication of the RCTs was not associated with an abrupt change in volume (IRR:0.943[0.824–1.079]; *P =* 0.393) but was significantly associated with a change in trend towards 2% fewer SAD surgeries on average per month (IRR:0.984[0.971–0.998]; *P* = 0.021), i.e. 18% fewer surgeries per year (0.984^12^). Within the control group, there was no significant pre-publication trend (IRR:1.000[0.992–1.007]; *P* = 0.939) and no significant association between publication of the RCTs with any changes in level (IRR:0.998[0.936–1.063]; *P* = 0.940) or trend (IRR:1.002[0.993–1.012]; *P* = 0.645) (Fig. 1).

**Fig. 2:**
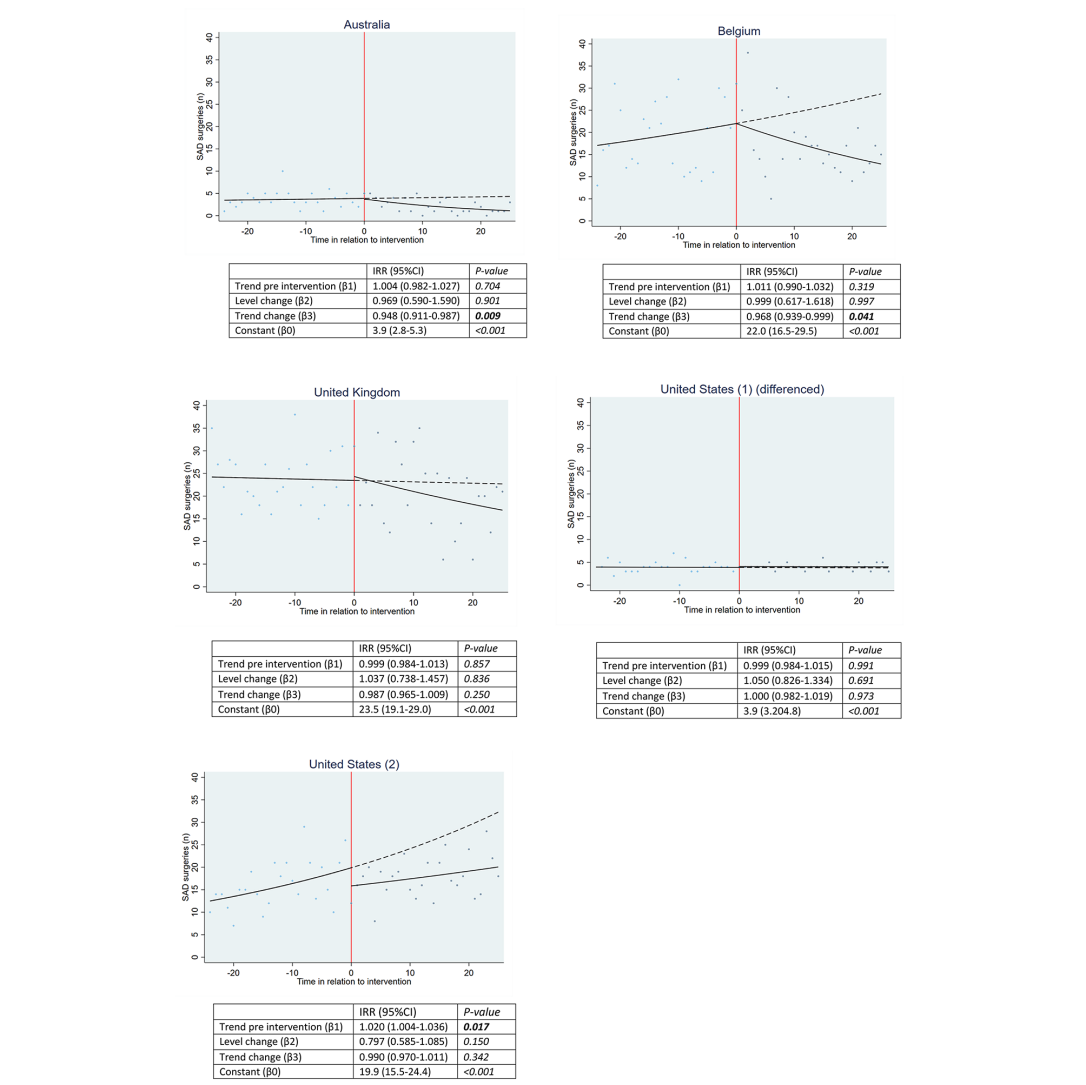
Outcomes SAPS group- individual hospitals. Shows the fitted trend lines and regression coefficients of the number of monthly SAD surgeries within the individual hospitals before and after publication of the RCTs (time in relation to intervention represented in *months*). The dashed lines represent the fitted trend lines post-intervention as if the RCTs had not been published. Significant values are presented in bold. Abbreviations: SAD = subacromial decompression, IRR = incidence rate ratio, 95%CI = 95% Confidence Interval

Given the wide variation in volumes of SAD surgeries (Table 1), we also examined the trends for individual hospitals as there may have been contrasting trends that could level out in an overall analysis (Fig. 2). This analysis showed that the association with a changing trend towards reduced volume of SAD surgeries was shown for 4 of 5 hospitals, albeit only significant in the Australian (IRR:0.948[0.911–0.987]; *P* = 0.009) and Belgium (IRR:0.968[0.939–0.999]; *P* = 0.041) hospitals. One US hospital showed a significantly increasing pre-publication trend (IRR:1.020[1.004–1.036]; *P* = 0.017) with publication of the RCTs not associated with any significant change in level or trend, i.e. it continued to increase. In the control group, volumes of procedures increased in the Australian hospital before publication of the RCTs (IRR:1.002[1.000-1.005]; *P* = 0.026). Publication of the RCTs was associated with a significant change in level (IRR:0.931[0.885–0.978]; *P* = 0.004), but not with a changing trend i.e. it continued to increase (IRR:1.001[0.988–1.004]; *P* = 0.406). No significant associations with changes in level and/or trend were found for the other hospitals (Appendix C).

### Potential shifts towards other procedures

**Fig. 3:**
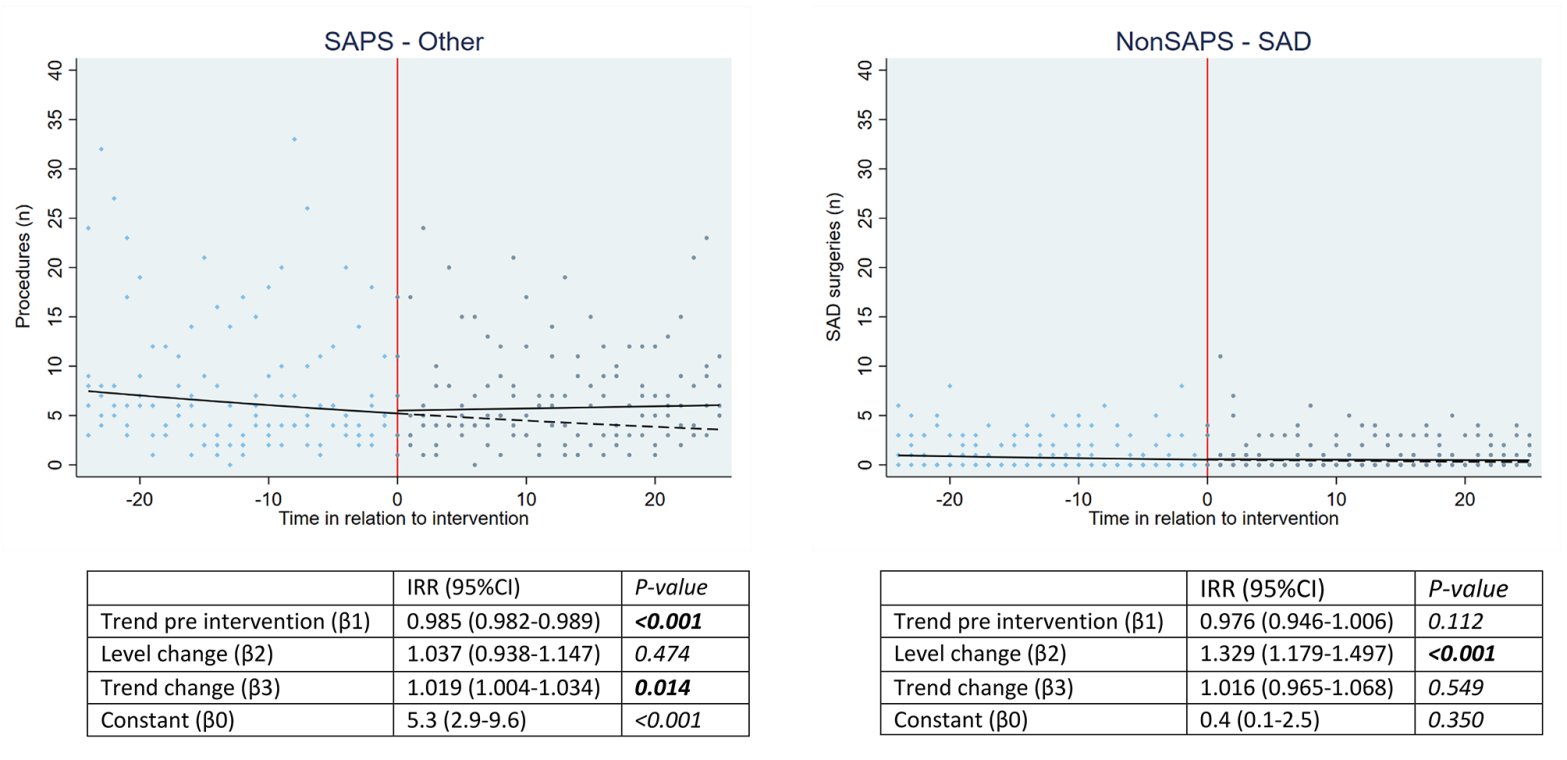
Outcomes SAPS-Other group and NonSAPS-SAD group. Shows the fitted trend lines and regression coefficients, adjusted for clustering of patients within hospitals, of the number of monthly procedures within the SAPS-Other group (left) and NonSAPS-SAD group (right) before and after publication of the RCTs (time in relation to intervention represented in *months*). The dashed lines represent the fitted trend lines post-intervention as if the RCTs had not been published. Significant values are presented in bold. Abbreviations: SAD = subacromial decompression, IRR = incidence rate ratio, 95%CI = 95% Confidence Interval

The results from the sensitivity analysis are shown in Fig. 3. Within the SAPS-Other group, there was a significantly decreasing overall trend of about 2% per month before publication of the RCTs (IRR:0.985[0.982–0.989]; *P* = < 0.001*).* Publication of the RCTs was not associated with a significant change in level (IRR:1.037[0.938–1.147]; *P* = 0.474*)* but was associated with significant increase of 2% per month in other procedures within SAPS patients (IRR:1.019[1.004–1.034]; *P* = 0.014). The most frequently performed procedures within the SAPS-Other group included repair of shoulder tendon, excision of shoulder tendon and replacement of the shoulder joint. When examining this further within individual hospitals, the association with an increased trend of other procedures within SAPS patients was seen in 4 of 5 hospitals, although significance was only reached in the UK (IRR:1.049[1.013–1.085]; *P* = 0.007) and one US hospital (IRR:1.031[1.001–1.063]; *P* = 0.042)(Appendix C).

Within the NonSAPS-SAD group, there was no significant overall pre-publication trend (Fig. 3). Publication of the RCTs was associated with a significant change in level (IRR:1.329[1.179–1.497]; *P* = < 0.001), but not with any significant changes in trend (Fig. 3). For individual hospitals, the numbers of performed procedures for the NonSAPS-SAD were low (data not shown).

## Discussion

The present study has shown that publication of the high-quality RCTs by Beard- and Paavola et al. in 2018[4, 6] was associated with a significantly reduced overall trend in use of SAD surgery of on average 2% per month (i.e. 18% per year), although the effect varied between hospitals. This association with a reduced trend in SAD surgery was shown for 4 of the 5 hospitals, albeit significant only in the Australian and Belgium hospitals, and was not seen in the control group. Sensitivity analysis showed that publication of the RCTs was also associated with a concurrent 2% monthly increased trend towards other procedures within SAPS patients and with an abrupt increase in volume of SAD surgeries in the Non-SAPS group.

The strength of the present study is that we used a controlled ITS design, a strong quasi-experimental design, that can estimate the effects of an intervention in a natural experimental setting with the control group taking into account any other interventions influencing the volume of surgeries [31]. Furthermore, all diagnosis and procedure data were harmonized to reconcile differences between coding systems. The hospitals were large academic centres, which provided a unique opportunity to evaluate the effect of evolving evidence on daily practice across different countries. Limitations of our study include the use of administrative data which could be subject to both over- or under-coding of patient characteristics such as more comorbidities, where for instance US hospitals may have higher occurrence of comorbidities due to financial incentives associated with coding. However, reimbursement of health services in Australia also depends on clinical coding, yet showed lower frequency of comorbidity in the current study. Particularly since we examined volumes of SAD surgery without adjusting for differences in patient-mix, this is unlikely to explain our results. Secondly, it is important to note that the study findings are only based on limited number of hospitals. As each centre was a large academic hospital, the included hospitals are broadly comparable but may differ from other(non-academic) hospitals in the selected countries, thus limiting the generalizability of our results to academic hospitals. Thirdly, no data on outpatient visits were available for analysis making it impossible to explore changes in the percentage of SAPS patients receiving SAD surgery. However, since the main outcome of interest was the volume of SAD surgeries which are performed as a day case surgery or require a hospital admission, it seems unlikely to have affected our results. Lastly, other interventions than the publication of the RCTs (e.g. payment policy- or guideline changes occurring around the same time) may have influenced clinician behaviour with regard to SAPS patients. However, we are unaware of other interventions during the period of interest and discussion among collaborating hospitals also did not suggest any simultaneous interventions.

### Comparison with literature

To our knowledge this is the first study that evaluates whether publication of the two placebo-controlled RCTs on treatment for SAPS in 2018 were associated with a change in existing trends in SAD surgery in hospitals from different countries. Various studies have investigated trends in earlier time periods when other RCTs showing on the effectiveness of SAD surgery were published [13, 18, 22]. A Finnish study reported a declining trend in volume of SAD surgery starting in 2007, but this was two years after the RCT by Haahr et al. [7] was published, so that it is unclear whether the decline was associated with publication of that RCT or something else. In the UK, a slight decrease in the number of SAD surgeries was observed after 2011/2012, two years after publication of the RCTs by Henkus- and Ketola et al. and also the starting year of the CSAW trial which eventually led to the publication by Beard in 2018 [4, 8, 9, 16]. A Scottish study found a decline in the use of SAD surgery starting in 2017, but this was one year before the RCT by Beard was published [22] and therefore unclear whether the decline is associated with publication of this RCT or due to the rising tide phenomenon [36]. Lastly, a decreasing trend was observed in the Netherlands, following a clinical practice guideline implementation in 2012 that advocated against SAD surgery, but lack of data for the period before guideline implementation made evaluation impossible [20]. Results of the present study therefore add to this literature that a change in trend is associated with publication of high-quality evidence.

Two studies describing decreasing trends in SAD surgeries showed a simultaneous increase in other procedures(e.g. rotator cuff surgery, acromioclavicular-joint excision), suggesting a shift in coding patterns [19, 22]. Our sensitivity analyses also showed that publication of the RCTs was associated not only with a change towards a reduced trend in use of SAD surgery in SAPS patients, but also with an increased trend in other procedures among SAPS patients, and an abrupt increase in the use of SAD surgery for Non-SAPS patients. Therefore, only evaluating the total number of SAD surgeries could create a distorted picture how research results affect daily practice, if a decline of a surgical procedure is accompanied by a shift in coding practices rather than not performing the procedure at all.

### Interpretation and clinical implications

The results of this study suggest that publication of high-quality RCTs can change clinical practice. Even though statistical significance does not equal clinical relevance, we believe our results are relevant because of the strong recommendation against the use of SAD surgery for SAPS, so that every reduction in the use of this low-value care procedure is important. However, we cannot rule out the possibility that there has been a concurrent shift in coding practice given that publication of the RCTs was associated both with an overall 2% reduction in trend in SAD surgeries but also a 2% increase in other procedures among SAPS patients. Rather than a reduction of care providing no benefit for patients, it may indicate substitution towards other surgical procedures. The use of a control group provided stronger evidence to support the publication of the RCTs really causing the observed changes in trends. We also showed large variation in effect between hospitals from various countries, suggesting that the uptake of evolving evidence differs significantly between healthcare providers potentially influenced by different reimbursement for healthcare services. Additionally, SAPS is an umbrella diagnosis, covering a large heterogeneous group of shoulder problems with unknown aetiology and despite high-quality evidence showing no benefit of SAD surgery for SAPS patients, clinical guidelines remain unclear on the best alternative(non-surgical) treatment [14]. This leaves the clinicians with uncertainty about the best alternative treatment and might introduce action bias, the general preference for active over passive treatment in clinical decision-making [37, 38]. All of these factors highlight the complexities of implementing such international recommendations in daily practice even if based on strong evidence, and more research is needed to understand which factors influence the uptake of evidence to change clinical practice towards reducing low-value care and to improve quality of care.

The presented case of SAD surgery for SAPS can be viewed as an example to explore the relationship between evolving evidence and changes in clinical practice in various countries. Similar study designs can be used to evaluate and monitor the effect of clinical guidelines or research evidence on daily practice for other procedures considered to have no or little benefit for patients. Reducing low-value care is of vital importance to protect patients from harm and to lower the financial burden on healthcare systems. International campaigns have been launched that aim to improve the quality of care by reducing low-value care. Quick dissemination of new evidence into clinical practice is in line with these international campaigns and can be done in the context of collaboratives, which are considered an effective approach to shared learning and improvements in the quality of care [39]. Our results illustrate the value of such collaboratives to compare clinical practice and to use observed variation as a starting point to enable improvements in quality of care.

## Electronic supplementary material

Below is the link to the electronic supplementary material.

Supplementary Material 1: Appendix A. Hospitals participating in the Global Health Data @ Work (GHD@Work) collaborative.

Supplementary Material 2: Appendix B. Used diagnosis & procedure codes and CCS groups.

Supplementary Material 3: Appendix C. Additional results.

## Data Availability

All data generated or analysed during this study are included in this published article. Formal permission to use the data in the Global Health Data@work projects was obtained from all participants. The datasets are only available for Global Health Data@work participants.

## List of abbreviations

RCT: Randomised Controlled Trial
SAD: Subacromial Decompression Surgery
SAPS: Subacromial Pain Syndrome
ITS: Interrupted Time Series
GHD@Work: Global Health Data@Work
MSK: Musculoskeletal
CCS: Clinical Classification System
SD: Standard Deviation
CI: Confidence Interval
IRR: Incidence Rate Ratio
