# Supplementary material for: The relationship between publication of high-quality evidence and changes in the volume and trend of subacromial decompression surgery for patients with subacromial pain syndrome in hospitals across Australia, Europe and the United States: a controlled interrupted time series analysis
Source: BMC Musculoskelet Disord. 2023 Jun 3;24:456. doi: 10.1186/s12891-023-06577-6 (PMC10239046; doi:10.1186/s12891-023-06577-6)
Supplement: Supplementary file 1 — Supplementary Material 1: Appendix A. Hospitals participating in the Global Health Data @ Work (GHD@Work) collaborative. [file 12891_2023_6577_MOESM1_ESM.docx]

**APPENDIX A:** Hospitals participating in the Global Health Data @ Work (GHD@Work) collaborative.

| Country | Hospital |
| --- | --- |
| *Australia* | Alfred Health Hospital Melbourne |
| *Belgium* | University Hospital Leuven |
| *The Netherlands* | Leiden University Medical Centre (LUMC) |
| *United Kingdom* | University Hospital Coventry |
| *United States (1)* | Keck Medical Center of the University of Southern California |
| *United States (2)* | Hackensack Meridian Health |
