# Supplementary material for: The relationship between publication of high-quality evidence and changes in the volume and trend of subacromial decompression surgery for patients with subacromial pain syndrome in hospitals across Australia, Europe and the United States: a controlled interrupted time series analysis
Source: BMC Musculoskelet Disord. 2023 Jun 3;24:456. doi: 10.1186/s12891-023-06577-6 (PMC10239046; doi:10.1186/s12891-023-06577-6)
Supplement: Supplementary file 2 — Supplementary Material 2: Appendix B. Used diagnosis & procedure codes and CCS groups. [file 12891_2023_6577_MOESM2_ESM.docx]

**Appendix C. Additional results.**

1. **Regression coefficients of the control group per health care provider.**

|  | **Australia** | | **Belgium** | | **United Kingdom** | | **United States (1) (differenced)** | | **United States (2)**  **(differenced)** | |
| --- | --- | --- | --- | --- | --- | --- | --- | --- | --- | --- |
|  | IRR (95%CI) | *P-value* | IRR (95%CI) | *P-value* | IRR (95%CI) | *P-value* | IRR (95%CI) | *P-value* | IRR (95%CI) | *P-value* |
| Trend pre intervention (β1) | 1.002  (1.000-1.005) | ***0.026*** | 1.001  (0.997-1.006) | *0.507* | 1.002  (0.999-1.005) | *0.177* | 1.002  (0.971-1.034) | *0.909* | 0.995  (0.967-1.023) | *0.719* |
| Level change (β2) | 0.931  (0.885-0.978) | ***0.004*** | 1.002  (0.920-1.092) | *0.960* | 1.036  (0.969-1.108) | *0.300* | 1.053  (0.534-2.078) | *0.881* | 0.992  (0.577-1.705) | *0.976* |
| Trend change (β3) | 1.001  (0.988-1.004) | *0.406* | 0.997  (0.992-1.003) | *0.300* | 0.996  (0.992-1.000) | *0.072* | 0.998  (0.957-1.040) | *0.920* | 1.009  (0.972-1.048) | *0.649* |
| Constant (β0) | 1507  (1453-1562) | *<0.001* | 1042  (976-1113) | *<0.001* | 1345  (1276-1417) | *<0.001* | 179  (106-303) | *<0.001* | 106  (75-150) | *<0.001* |

1. **Regression coefficients of the SAPS-Other group per health care provider.**

|  | **Australia** | | **Belgium** | | **United Kingdom** | | **United States (1)** | | **United States (2)** | |
| --- | --- | --- | --- | --- | --- | --- | --- | --- | --- | --- |
|  | IRR (95%CI) | *P-value* | IRR (95%CI) | *P-value* | IRR (95%CI) | *P-value* | IRR (95%CI) | *P-value* | IRR (95%CI) | *P-value* |
| Trend pre intervention (β1) | 0.994  (0.969-1.020) | *0.636* | 0.987  (0.970-1.004) | *0.124* | 0.979  (0.956-1.004) | *0.099* | 0.980  (0.955-1.006) | *0.137* | 0.983  (0.956-1.010) | *0.205* |
| Level change (β2) | 0.995  (0.581-1.704) | *0.985* | 1.032  (0.725-1.469) | *0.863* | 0.997  (0.556-1.787) | *0.992* | 1.304  (0.847-2.009) | *0.228* | 0.884  (0.479-1.633) | *0.695* |
| Trend change (β3 | 0.996  (0.958-1.035) | *0.819* | 1.008  (0.983-1.034) | *0.535* | 1.049  (1.013-1.085) | ***0.007*** | 1.031  (1.001-1.063) | ***0.042*** | 1.030  (0.990-1.071) | *0.141* |
| Constant (β0) | 3.822  (1.568-3.780) | *<0.001* | 15.389  (12.063-19.631) | *<0.001* | 2.867  (1.928-4.264) | *<0.001* | 4.839  (3.433-6.819) | *<0.001* | 4.495  (2.874-7.031) | *<0.001* |
