# Supplementary material for: The relationship between publication of high-quality evidence and changes in the volume and trend of subacromial decompression surgery for patients with subacromial pain syndrome in hospitals across Australia, Europe and the United States: a controlled interrupted time series analysis
Source: BMC Musculoskelet Disord. 2023 Jun 3;24:456. doi: 10.1186/s12891-023-06577-6 (PMC10239046; doi:10.1186/s12891-023-06577-6)
Supplement: Supplementary file 3 — Supplementary Material 3: Appendix C. Additional results. [file 12891_2023_6577_MOESM3_ESM.docx]

**Appendix B: Used diagnosis & procedure codes and CCS groups.**

**1. Used diagnosis and procedure codes to identify subacromial decompression (SAD) surgery per coding system.**

*Used diagnosis codes:*

| ICD-10 Code | *Description* |
| --- | --- |
| **M75.1** | Rotator cuff syndrome |
| **M75.2** | Bicipital tendinitis |
| **M75.3** | Calcific tendinitis of shoulder |
| **M75.4** | Impingement syndrome of shoulder |
| **M75.5** | Bursitis of shoulder |

*In combination with any of the following procedure codes:*

*A. ACHI (Australia)*


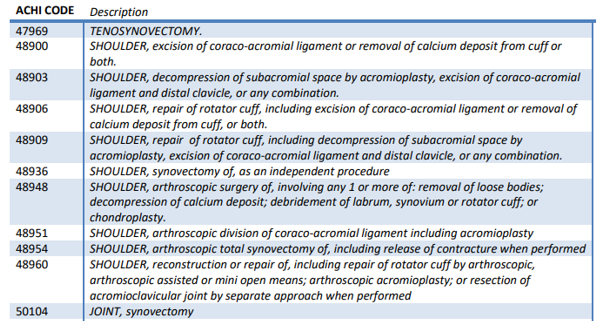


*B. ICD10-PCS (Belgium and United States)*


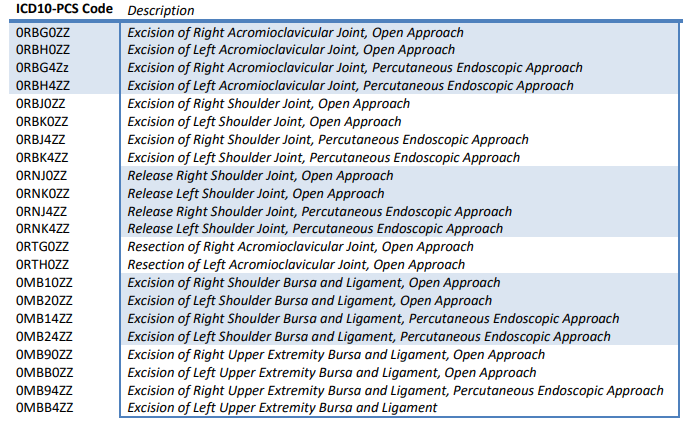


*C. OPCS (United Kingdom)*


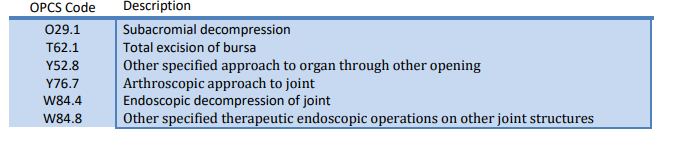


*D. CBV (the Netherlands)*


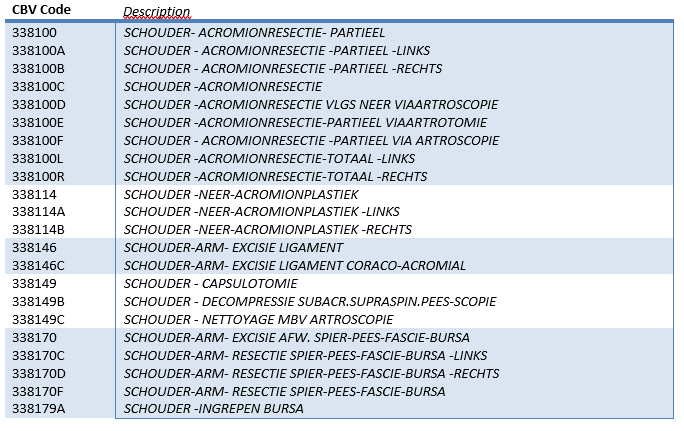


**2. CCS groups within the ICD-10 clusters ‘Diseases of the musculoskeletal system and connective tissue disease’ & ‘Injury, poisoning and certain other consequences of external causes’ (“MSK Clusters”)**

| *ICD-10 Cluster* | *CCS Group* | *Description* |
| --- | --- | --- |
| *Diseases of the musculoskeletal system and connective tissue disease* | *54* | *Gout and other crystal arthropathies* |
|  | *201* | *Infective arthritis and osteomyelitis (except that caused by tuberculosis or sexually transmitted disease)* |
|  | *202* | *Rheumatoid arthritis and related disease* |
|  | *203* | *Osteoarthritis* |
|  | *204* | *Other non-traumatic joint disorders* |
|  | *205* | *Spondylosis; intervertebral disc disorders; other back problems* |
|  | *206* | *Osteoporosis* |
|  | *207* | *Pathological fracture* |
|  | *208* | *Acquired foot deformities* |
|  | *209* | *Other acquired deformities* |
|  | *210* | *Systemic lupus erythematosus and connective tissue disorders* |
|  | *211* | *Other connective tissue disease* |
|  | *212* | *Other bone disease and musculoskeletal deformities* |
| *Injury, poisoning and certain other consequences of external causes* | *225* | *Joint disorders and dislocations; trauma-related* |
|  | *226* | *Fracture of neck of femur (hip)* |
|  | *227* | *Spinal cord injury* |
|  | *228* | *Skull and face fractures* |
|  | *229* | *Fracture of upper limb* |
|  | *230* | *Fracture of lower limb* |
|  | *231* | *Other fractures* |
|  | *232* | *Sprains and strains* |
|  | *233* | *Intracranial injury* |
|  | *234* | *Crushing injury or internal injury* |
|  | *235* | *Open wounds of head; neck; and trunk* |
|  | *236* | *Open wounds of extremities* |
|  | *237* | *Complication of device; implant or graft* |
|  | *238* | *Complications of surgical procedures or medical care* |
|  | *239* | *Superficial injury; contusion* |
|  | *240* | *Burns* |
|  | *241* | *Poisoning by psychotropic agents* |
|  | *242* | *Poisoning by other medications and drugs* |
|  | *243* | *Poisoning by nonmedicinal substances* |
|  | *244* | *Other injuries and conditions due to external causes* |
